# Neonatal and Two-Year Prognosis of Eutrophic Newborns from Monochorionic Diamniotic Twin Pregnancies Complicated by Selective Intrauterine Growth Restriction

**DOI:** 10.3390/children12050615

**Published:** 2025-05-08

**Authors:** Marie-Anne Jarry, Nayri Topalian, Lauréline Cosnard, Claude D’Ercole, Cécile Chau, Barthélémy Tosello

**Affiliations:** 1 Department of Neonatal Medicine, North Hospital, Assistance Publique-Hôpitaux de Marseille, 13015 Marseille, France; marie-anne.jarry@ap-hm.fr (M.-A.J.); nayri.topalian1@ap-hm.fr (N.T.); 2 Department of Gynecology and Obstetrics, North Hospital, Assistance Publique-Hôpitaux de Marseille, 13015 Marseille, France; laureline.cosnard@ap-hm.fr (L.C.); claude.dercole@ap-hm.fr (C.D.); cecile.chau@ap-hm.fr (C.C.); 3 EA3279, CEReSS, Health Service Research and Quality of Life Center, Aix-Marseille Université, 13284 Marseille, France; 4 CNRS, EFS, ADES, Aix-Marseille Université, 13007 Marseille, France

**Keywords:** monochorionic pregnancies, morbidity, neurodevelopment, preterm birth, twins

## Abstract

Background: Monochorionic diamniotic (MCDA) twin pregnancies are at risk of complications, particularly selective intrauterine growth restriction. The objective of this study was to evaluate the two-year neurologic outcomes of the eutrophic newborns from monochorionic diamniotic twin pregnancies who were complicated by selective intrauterine growth restriction, compared to newborns from uncomplicated MCDA pregnancies. Our hypothesis was to determine whether selective IUGR in these pregnancies was specifically associated with a risk of delayed psychomotor development at two years old. Methods: We conducted a retrospective–prospective observational cohort study of children from pregnancies and deliveries which were monitored at Hospital Nord of Marseille between 2012 and 2021. The primary outcome measure was the comparison of the Ages and Stages Questionnaire (ASQ) scores at the age of two years between the two groups. The secondary outcome measure was a composite score including the following: neonatal death, grade III or IV intraventricular hemorrhage (IVH) at cerebral MRI or cranial ultrasound, periventricular leucomalacia (PVL) at brain MRI, bronchopulmonary dysplasia (BPD), and necrotizing enterocolitis (NEC) of stages II or III. Results: A total of 57 eutrophic children were included in the group from monochorionic twin pregnancies complicated by selective IUGR and 270 children in the group from MCDA twin pregnancies with no complications. The composite morbidity and mortality criterion, including neonatal death, grade III or IV IVH, the presence of PVL, BPD, and/or stage II or III NEC, was 11% in eutrophic newborns from the MCDA group with IUGR and 5% in the uncomplicated MCDA group, with no statistically significant difference (*p* = 0.18). The 2-year follow-up allowed for the comparison of a total of 38 eutrophic children from complicated pregnancies and 134 children from uncomplicated pregnancies. The median ASQ score at 24 months was 255 in the complicated pregnancy group and 240 in the uncomplicated pregnancy group, with no statistically significant difference (*p* = 0.27) after adjustment. Conclusions: Our study did not show a statistically significant difference in the neurodevelopmental follow-up of eutrophic children from monochorionic diamniotic twin pregnancies with selective intrauterine growth restriction compared to newborns from the same pregnancies without complications.

## 1. Introduction

Monochorionic diamniotic (MCDA) twin pregnancies represent 10 to 15% of twin pregnancies [1], which correspond to approximately 1500 births per year in France. It is now generally recognized that monochorionic twin pregnancies have a higher morbidity and mortality rate compared to dichorionic pregnancies [2]. MCDA twin pregnancies are associated with specific complications (twin–twin transfusion syndrome (TTTS), selective intrauterine growth restrictions, twin anemia polycythemia sequences (TAPS), and acute feto-fetal hemorrhages subsequent to a single intrauterine fetal death) [3,4,5,6]. Over the long term, children born from twin pregnancies have an increased risk of neurological morbidity compared to singleton births [7,8,9]. Compared to bichorionic diamniotic (BCDA) pregnancies, MCDA pregnancies are likely to deliver more preterm and lower-birth-weight babies and to display an excess of neonatal morbidity and mortality (death, intraventricular hemorrhage (IVH) grade III or IV, necrotizing enterocolitis (NEC), and neonatal anemia) [10,11,12,13,14]. At 2 years, neurodevelopmental difficulties are more marked in MCDA twins compared to BCDA twins [12,14,15].

These MCDA twin pregnancies are subject to specific complications, which may be related to an unequal distribution of placental circulation [16,17], a velamentous cord insertion [18,19], and the presence of placental vascular anastomoses [20]. These placental vascular anastomoses can be arteriovenous, venovenous, or arterioarterial, in varying proportions, with diverse pathophysiological implications [21,22,23].

Placental vascular anastomoses are directly responsible for complications such as twin-to-twin transfusion syndrome (TTTS), twin anemia–polycythemia sequence (TAPS), and even twin reversed arterial perfusion sequence (TRAPS), which themselves result in intrauterine growth discordance [21]. These complications are independent risk factors for adverse neurological outcomes in newborns and children [24,25,26]. Moreover, there is a pathophysiological continuum between these complications and selective intrauterine growth restriction (sIUGR) [18].

It has been demonstrated that MCDA twin pregnancies have an increased risk of developing selective intrauterine growth restriction (sIUGR) [21,27], as well as a higher risk of preeclampsia and induced or spontaneous preterm birth [28] compared to dichorionic pregnancies. Selective IUGR affects 10 to 15% of MCDA twin pregnancies [1] and is the second cause of perinatal morbidity and mortality, after prematurity.

The definition of selective intrauterine growth restriction (sIUGR) was established by an expert consensus in 2019 by Khalil et al. [29]. It is defined when either one twin has an estimated fetal weight (EFW) below the 3rd percentile or when at least two of the following four criteria are present: an EFW of one twin below the 10th percentile, an abdominal circumference below the 10th percentile, an estimated weight discordance of ≥25%, and an umbilical artery pulsatility index of the smaller twin above the 95th percentile.

The follow-up of these pregnancies involves the regular assessment of fetal growth, the analysis of fetal heart rate patterns, and umbilical Doppler studies. Doppler ultrasound examination allows for the frequent evaluation of umbilical blood flow and classifies it into three categories according to Gratacós’ classification, which is associated with prognostic values for survival and neurological outcomes [23].

It has been demonstrated that eutrophic newborns from MCDA twin pregnancies with sIUGR are at a higher risk of cerebral injury compared to their twin sibling [30,31,32,33] and have an increased risk of neonatal morbidity [6].

On the contrary, according to a 2022 meta-analysis [34], in DCBA twin pregnancies, the twin affected by IUGR would be at a higher risk of psychomotor developmental delay than their eutrophic twin sibling [35].

Although the neurological outcomes of hypotrophic infants from these pregnancies have been studied [36], no research, to our knowledge, has assessed the long-term neurological development of eutrophic newborns after neonatal care, in comparison to those from uncomplicated pregnancies [34]. Identifying a less favorable neurological prognosis in these children could support the need for closer obstetric monitoring (ultrasound and Doppler) and a delivery date scheduled according to this monitoring. It would also allow more frequent and extended pediatric follow-up for these infants.

The aim of our study was to assess psychomotor development at the age of two years in these children, compared to those from uncomplicated MCDA twin pregnancies. Our hypothesis was to determine whether selective IUGR in these pregnancies was specifically associated with a risk of delayed psychomotor development at two years old.

## 2. Materials and Methods

We conducted a single-center, retrospective–prospective cohort observational study. The study was carried out in the Departments of Gynecology-Obstetrics, Maternity, and Neonatology at the Marseille University Hospital Center (AP-HM), specifically at the Hôpital Nord de Marseille, located in the Provence-Alpes-Côte d’Azur region.

### 2.1. Study Population

Eutrophic Group from MCDA Twin Pregnancies with selective IUGR (sIUGR): the included children were alive eutrophic infants from MCDA twin pregnancies complicated by the sIUGR of the other twin. Their pregnancy follow-up and delivery took place at Hôpital Nord de Marseille, and they were born alive between 1 January 2012 and 31 December 2021.

Selective intrauterine growth restriction (sIUGR) was defined in our study according to the criteria established by Khalil et al. [29]. This included an estimated fetal weight (EFW) of either twin below the 3rd percentile or at least two of the following four criteria: an EFW of either twin below the 10th percentile, an abdominal circumference of one of the twins below the 10th percentile, an estimated weight discordance of ≥25%, and an umbilical artery pulsatility index of the smaller twin above the 95th percentile.

Group from MCDA Twin Pregnancies Without Complications: the children included in this group were live-born infants from MCDA twin pregnancies without complications, whose prenatal follow-up and delivery took place at Hôpital Nord de Marseille, born between 1 January 2012 and 31 December 2021.

The exclusion criteria for both groups were MCDA twin pregnancies complicated by TTTS, TRAPS, and TAPS, intrauterine growth restriction (IUGR) affecting both fetuses, and fetal malformations.

### 2.2. Study Objectives

The primary outcome of our study was to compare the neurodevelopmental status, assessed using the Ages and Stages Questionnaire (ASQ) at the age of 2 years, between eutrophic children from MCDA twin pregnancies with IUGR and children from uncomplicated MCDA twin pregnancies.

The secondary objectives were to assess and compare neonatal morbidity and mortality between the two groups.

### 2.3. Perinatal and Neonatal Data

Obstetric management followed the current guidelines of the French National College of Obstetricians and Gynecologists throughout the study period and was comparable between the two groups [37].

Neonatal care also followed the current guidelines of the French Society of Neonatology. Diagnostic tests (electroencephalogram, fundoscopy, transfontanellar ultrasound, and brain MRI) were performed based on gestational age, birth weight, and adaptation to extrauterine life, following the protocols of the Mediterranean Perinatal Network (Réseau de périnatalité PACA Corse Monaco). These tests were performed similarly in both groups.

The assessment of hemorrhagic lesions on transfontanellar ultrasound was performed according to Papile’s classification.

Bronchopulmonary dysplasia was defined as the need for oxygen therapy or ventilatory support at 36 weeks of corrected gestational age and/or 28 days of life.

The presence and grading of NEC were determined and classified based on the modified Bell’s staging criteria.

Our secondary outcome measure was a composite score including the following: neonatal death, grade III or IV intraventricular hemorrhage (IVH) detected on brain MRI or cranial ultrasound, periventricular leukomalacia (PVL) on brain MRI, bronchopulmonary dysplasia (BPD), and stage II or III necrotizing enterocolitis (NEC) [38,39,40,41].

The collection of obstetric and neonatal data was carried out retrospectively from medical records, in a comparable manner between the two groups.

### 2.4. Development at 2 Years

The Ages and Stages Questionnaire (ASQ), developed in 1997, is an assessment measure designed to evaluate psychomotor development in children from 4 months to 5 years of age [42]. Among its 19 different questionnaires, the ASQ-24 is designed specifically to assess development progress at 24 months, with an assessment period ranging from 23 months to 25 months and 15 days, considering corrected age in cases of prematurity. The third version of the ASQ-24, updated and published in 2009, was used in this study, with a validated scoring system applicable to both medical and parental assessments. This questionnaire evaluates five key developmental domains: communication, gross motor skills, fine motor skills, problem-solving abilities, and personal–social skills. Each domain is assessed through six questions, with responses scored on a 10-point scale using three possible ratings: 10 (yes), 5 (sometimes), and 0 (not yet). The total score, reflecting the child’s overall development, is calculated out of 300 points, representing the maximum possible score.

A pathological score is identified as a score equal to or lower than −2 standard deviations (SDs) from the mean in at least one domain. A score below 220 suggests that at least one domain falls below −1 SD, indicating the need for closer monitoring.

Additional questions help detect underlying neurosensory abnormalities (hearing loss or myopia, etc.) that may affect the score outcome.

The ASQ score is a questionnaire routinely administered by doctors to follow up children who are part of the Mediterranean Perinatal Network across the Provence-Alpes-Côte d’Azur, Corsica, and Monaco regions.

Data collection at 2 years of age was conducted prospectively from May 2023 to February 2024 for children aged between 23 months and 25 months and 15 days during this period. For all other children, data collection was performed retrospectively. Information was gathered from medical follow-up records when available, and when unavailable, through parental interviews.

### 2.5. Statistical Analysis

All statistical analyses were performed using R (version 4.2.2). To identify significant differences between the two groups, linear models (generalized for categorical variables) were used, adjusted for term, weight, and sex. Given the relatively small sample size and the non-normality of model residuals, permutation tests were subsequently applied (using the R package).

Maternal, obstetric, and delivery characteristics were compared between the two groups. Results are presented as *p*-values, with statistical significance defined as *p* ≤ 0.05.

Due to the significant number of participants lost to follow-up between inclusion and the 2-year analysis, a new comparison of maternal, obstetric, and delivery characteristics was conducted. Results are presented as *p*-values, with statistical significance defined as *p* ≤ 0.05.

## 3. Results

### 3.1. Perinatal Characteristics of the Population

A total of 264 MCDA twin pregnancies were included, based on delivery data from Hôpital Nord de Marseille between 1 January 2012 and 31 December 2024. In total, 72 pregnancies met the exclusion criteria and were therefore removed from the study: 31 cases of TTTS, 12 cases of TAPS, 25 pregnancies treated with laser therapy, and 4 pregnancies with IUGR affecting both twins.

**Figure 1:**
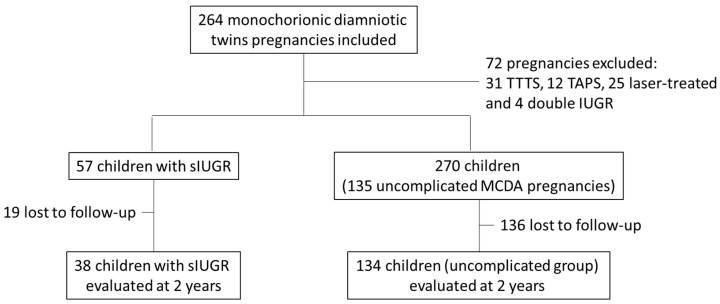
Flow chart. MCDA: monochorionic diamniotic; IUGR: intrauterine growth restriction; sIUGR: selective intrauterine growth restriction; TTTS: twin-to-twin transfusion syndrome; TAPS: twin anemia–polycythemia sequence.

The remaining 192 pregnancies were divided into two groups based on the inclusion criteria: 57 eutrophic twins from MCDA twin pregnancies with IUGR and 270 twins (135 pregnancies) from the same type of pregnancies without complications (Figure 1).

Obstetric, delivery, and neonatal data from the initial population of both groups were compared. Antenatal demographic and obstetric data were similar between the two groups (Table 1).

The median maternal age at pregnancy was approximately 30 years. The maternal body mass index (BMI) was 22.4 kg/m^2^ for eutrophic twins from MCDA twin pregnancies with IUGR and 24.1 kg/m^2^ for those from uncomplicated GG MCDA pregnancies. Gravidity and parity were comparable between the two groups, and fetal sex distribution was balanced.

A significant difference was observed in birth methods, with 84% of births by cesarean section in MCDA twin pregnancies with IUGR compared to 50% in uncomplicated MCDA twin pregnancies. Spontaneous birth was the most common delivery mode in uncomplicated MCDA twin pregnancies (62 out of 135 pregnancies) but less frequent in MCDA twin pregnancies with IUGR (13 out of 57 pregnancies).

The gestational age was statistically lower in MCDA twin pregnancies with IUGR compared to uncomplicated MCDA twin pregnancies, at 33 weeks and 36 weeks, respectively. Likely linked to the difference in gestational age between the groups, anthropometric data on birth weight, head circumference, and length at birth were numerically lower in MCDA with IUGR than in newborns from uncomplicated MCDA. The average birth weight was 1914 g and 2306 g, respectively.

### 3.2. Neonatal Assessment

The occurrence of one or more components of the composite morbidity and mortality criterion—including neonatal death, grade III or IV IVH, the presence of PVL, BPD, and/or stage II or III NEC—was 11% in eutrophic newborns from MCDA with IUGR and 5% in those from uncomplicated MCDA pregnancies. This difference was not statistically significant (*p* = 0.18) (Table 2).

The proportion of patients presenting abnormalities on brain MRI was higher in MCDA twin pregnancies with IUGR than in uncomplicated MCDA pregnancies, with 56% and 39% of those undergoing brain imaging by MRI, respectively. However, this difference was not statistically significant (*p* = 0.32). The proportion of patients with periventricular leukomalacia was significantly higher in MCDA twin pregnancies with IUGR, with two affected patients (13% of those who underwent brain MRI), while no cases were observed in uncomplicated MCDA twin pregnancies (*p* = 0.03). Higher proportions of IVH and ventricular dilations were also observed in the MCDA twin pregnancies with IUGR group, at 6% and 13%, respectively, compared to 4% and 4% in uncomplicated MCDA twin pregnancies. However, these differences were not statistically significant.

The percentage of intensive care unit hospitalizations was higher among newborns from MCDA twin pregnancies with IUGR, with 63% requiring hospitalization compared to 24% in uncomplicated MCDA pregnancies (*p* < 0.001). The length of hospital stay did not differ between the two groups.

A significantly higher proportion of ventilatory support was also observed in eutrophic patients from the MCDA twin pregnancies with IUGR group, with 68% compared to 26% in uncomplicated MCDA pregnancies (*p* < 0.001). Additionally, the proportion of patients diagnosed with hyaline membrane disease was higher in MCDA pregnancies with IUGR, affecting 58% of patients versus 24% in the uncomplicated MCDA group (*p* < 0.001).

### 3.3. Neurodevelopmental Assessment at 2 Years

The final population that completed the ASQ at 2 years consisted of 38 patients from the eutrophic MCDA twin pregnancies with IUGR group and 134 children from the uncomplicated MCDA twin pregnancies group. The perinatal data of the final 2-year population were compared (Table 3).

The median ASQ score at 24 months was 252 in the MCDA with IUGR group and 240 in the uncomplicated MCDA group (Table 4), with no statistically significant difference (*p* = 0.26 after adjustment for confounding factors, including gestational age, birth weight, and sex).

The proportion of children with a score below 220 and/or at least one impaired domain was 29% in the MCDA twin pregnancies with IUGR group and 35% in the uncomplicated MCDA group, with no statistically significant difference (*p* = 0.51).

The most frequently affected domains in both groups were communication and social skills. The proportion of children with fine motor impairment at 2 years was 3% in the MCDA twin pregnancies with IUGR group and 4% in the uncomplicated MCDA group, with no statistically significant difference (*p* = 0.44).

An analysis of the ASQ by gestational age group was conducted, with a median ASQ score of 250 for children born between 26 and 31 weeks, 245 for those born between 32 and 34 weeks, and 260 for those born after 35 weeks. In the group of children from uncomplicated MCDA twin pregnancies, the median ASQ score was 240 for those born between 26 and 31 weeks, 232 for those born between 32 and 34 weeks, and 240 for those born after 35 weeks. The comparison of ASQ scores between gestational age subgroups did not reveal any significant differences (Table 5).

A comparison of ASQ scores based on the type of umbilical artery diastolic flow before birth in the IUGR twin was conducted, showing no statistically significant difference between the groups.

### 3.4. Comparison Between the Eutrophic Twin and the IUGR Co-Twin

#### Neonatal Data

The neonatal data of both infants from MCDA twin pregnancies with IUGR were compared. A significant difference was observed in umbilical cord blood pH at birth, with a pH of 7.34 in the eutrophic newborn and 7.30 in the IUGR newborn (*p* = 0.002). No statistically significant differences were found in the Apgar score or umbilical cord blood lactate levels.

The occurrence of the composite morbidity and mortality criterion was not significantly different between the two co-twins, with 11% in the eutrophic twin and 16% in the IUGR twin (*p* = 0.58). The comparison of neonatal data between the two co-twins revealed a significant difference in brain lesions on MRI, with 9 out of 16 patients who underwent brain MRI (56%) in the eutrophic twin group, compared to 3 out of 18 patients (17%) in the IUGR twin group (*p* = 0.001). Among the eutrophic patients who underwent brain MRI, 6% (one patient) had grade III or IV IVH, 13% (two patients) had periventricular leukomalacia, and 13% (two patients) had ventricular dilation. Other neonatal data were comparable between the two groups (Table 6).

### 3.5. ASQ at 2 Years

The ASQ score at 2 years was not significantly different between the two co-twins, with a median score of 255 in eutrophic twins and 245 in IUGR twins (*p* = 0.53). A total of 21% of eutrophic twins had at least one affected domain, compared to 37% of IUGR twins (*p* = 0.38). The most frequently affected domains were communication and social skills (Table 7).

## 4. Discussion

Our study found no statistically significant difference in the ASQ neurodevelopmental score at 24 months between eutrophic children from MCDA twin pregnancies with IUGR and those from uncomplicated MCDA pregnancies. This finding could help reassure parents during pregnancy about their child’s future psychomotor development. However, further studies are needed to confirm these results, including research assessing long-term neurological outcomes.

Previously, Hoarau et al. [43] demonstrated that chorionicity had no impact on psychomotor development at 5.5 years in children born prematurely (between 22 and 34 weeks’ gestation) within the French EPIPAGE-2 cohort, which included 1700 children, 480 of whom were from monochorionic twin pregnancies. Using the same cohort, Tosello et al. showed in 2021 that monochorionic twin pregnancies carried a higher risk of complications but that chorionicity did not have a negative impact on neonatal outcomes in twins admitted to the neonatal intensive care unit [44].

In 2021, Mercier et al. found no difference in the neurodevelopmental outcomes at 2 years between children from MCDA twin pregnancies with IUGR and singleton children with IUGR. To our knowledge, few authors have studied the long-term follow-up of the eutrophic twin in MCDA twin pregnancies complicated by IUGR [36].

Secondary analyses revealed a higher rate of neonatal morbidity and mortality in the eutrophic twin from MCDA twin pregnancies with IUGR, although the difference was not statistically significant. Mortality rates (4% in the IUGR group and 3% in the uncomplicated MCDA group, *p* = 0.81) were comparable to those reported in the 2016 meta-analysis by Buca et al. [27].

It was not possible to assess psychomotor development at 2 years based on brain MRI abnormalities. This was due to the small number of children with imaging abnormalities and a significant number of lost-to-follow-up cases, resulting in insufficient statistical power for the analysis. However, it is likely that a statistical association exists between abnormal brain imaging and poor psychomotor development, which is consistent with findings previously reported in the literature [45,46,47].

Secondary analyses revealed that the eutrophic twin from MCDA twin pregnancies with IUGR had a statistically higher prevalence of brain lesions on MRI compared to its IUGR co-twin, aligning with existing data in the literature [31]. However, the ASQ assessment at 2 years showed no difference between the eutrophic twin and its IUGR co-twin.

The main strength of this study was the considerable number of pregnancies and, as a result, the number of children included, with a follow-up at 2 years, specifically focusing on monochorionic diamniotic twin pregnancies with selective intrauterine growth restriction. To our knowledge, this is the first study to compare the specific neurological outcomes of these children to those from the same type of pregnancy without complications.

The primary outcome measure of our study was based on a validated and standardized questionnaire, officially recognized by the American Academy of Pediatrics (AAP). Multiple versions of this questionnaire exist, adapted to assess psychomotor development from 4 months to 5 years [48,49,50].

Our study had several limitations. This observational, single-center cohort study included twins born over a ten-year period. Given the significant advancements in neonatology and imaging techniques over the past decade, particularly in ultrasound (Doppler and transfontanellar) and MRI, our results should be interpreted with caution. There is a potential center effect, as well as notable changes in medical practices, that may not be fully reflected in our study’s data. Additionally, data collection was not conducted blindly to pregnancy complications and, consequently, to the affected group.

Socioeconomic factors play a significant role in psychomotor development [51,52,53]. However, these factors were not considered in our study. Additionally, we encountered a high number of lost-to-follow-up cases, possibly linked to limited access to healthcare, which may have influenced our study’s results.

The definition of selective intrauterine growth restriction (IUGR) is not universally standardized. The criteria we used for inclusion in our study were based on an expert consensus published in 2019 [29]. A 2022 meta-analysis [54] applying this new definition found that nearly twice as many pregnancies were classified as affected compared to the previous definition, while morbidity and mortality rates remained similar to those of uncomplicated monochorionic pregnancies. As a result, this new definition may be less specific than the previous one, which could weaken the generalizability of our findings.

We encountered data loss, with a significant number of participants lost to follow-up between study inclusion and the completion of the questionnaire at two years, leading to a reduction in statistical power. However, the number of completed questionnaires remained sufficient to conduct the statistical analysis.

It is now well understood that the first thousand days, from pregnancy to a child’s second year of life, play a crucial role in both neurological development and mental health. Additionally, psychomotor development is not a fixed process; while some children may catch up on delays, others may develop learning difficulties later on. For this reason, the ongoing follow-up of these children would be valuable to identify later-emerging neurodevelopmental delays or potential developmental catch-up.

The ASQ at 24 months is a screening tool for detecting psychomotor development disorders rather than a diagnostic instrument. It helps establish closer monitoring for children with a lower score in one or more areas and allows for the application of measures aimed at helping them catch up before reaching school age.

## 5. Conclusions

Our study did not identify a statistically significant difference in neurodevelopmental outcomes between eutrophic infants from MCDA twin pregnancies with IUGR and those from uncomplicated MCDA twin pregnancies. Further prospective studies with close medical follow-up would be needed to confirm these findings, ensuring that parents receive the most reliable information possible. Additionally, research assessing long-term neurological outcomes is essential to provide a more comprehensive understanding of these children’s development.

## Figures and Tables

**Table 1:** Demographic, delivery, and neonatal characteristics of the group of eutrophic newborns from MCDA twin pregnancies with sIUGR (sIUGR Group) and the group of newborns from uncomplicated MCDA twin pregnancies (Uncomplicated Group).

	sIUGR Group	Uncomplicated Group	*p* Value
**Number of pregnancies, n**	**57**	**135**	
**Age, years, median (min–max)**	30.9 (20.2–44.3)	29.9 (18.7–44.3)	0.75
**Gravidity, n, median (min–max)**	2 (1–10)	2 (1–10)	0.92
**Parity, n, median (min–max)**	1 (0–4)	1 (0–8)	0.26
**BMI, kg/m^2^, median (min–max)**	22.4 (16.4–43.0)	24.1 (16.3–45.0)	0.08
**Fetal sex, n (%)**			
Male	28 (49%)	75 (56%)	0.27
Female	29 (51%)	60 (44%)	
**Antenatal corticosteroid therapy, n (%)**			
Not administered	11 (19%)	70 (52%)	<0.001
Incomplete	0 (0%)	4 (3%)	
Complete	46 (81%)	61 (45%)	
**Mode of delivery, n (%)**			
Vaginal delivery	9 (16%)	68 (50%)	<0.001
Cesarean section	48 (84%)	67 (50%)	
**Indication for delivery, n (%)**			
Spontaneous	13	62	
Abnormal fetal heart rate	24	3	
Doppler abnormalities	15	3	
Growth restriction	12	7	
Other	5	41	
**Gestational age at birth, weeks of amenorrhea, median (min–max)**	33 (25–38)	36 (24–39)	<0.001
**Number of newborns, n**	**114**	**270**	
**Birth weight, grams, mean ± SD**	1914 ± 473	2306 ± 576	<0.001
**Head circumference at birth, centimeters, mean ± SD**	29 ± 2	31 ± 3	0.001
**Birth length, centimeters, mean ± SD**	42 ± 3	44 ± 4	0.001
**Growth discordance at birth, %, mean ± SD**	26 ± 10.4	7.8 ± 5.3	<0.001
**Birth weight percentile, %, mean ± SD**	44.3 ± 32	38.3 ± 25	0.69
**Apgar score, median, (min–max)**			
1 min	9 (0–10)	10 (0–10)	0.24
5 min	9 (5–10)	10 (3–10)	0.06
10 min	10 (8–10)	10 (5–10)	0.003
**Apgar score < 7, n/N (%)**			
1 min	8/57 (14%)	41/270 (15%)	0.7
5 min	4/57 (7%)	14/270 (5%)	0.7
10 min	0/57 (0%)	1/270 (0.3%)	0.25
**pH at birth, median, (min–max)**	7.34 (7.2–7.4)	7.31 (7.0–7.5)	0.003
pH < 7.15, n/N (%)	0/49	16/258 (6%)	0.06
**Lactate levels at birth, mmol/L, median, (min–max)**	2.3 (1.3–6.5)	2.9 (1.0–10.0)	0.06

**Table 2:** Neonatal outcomes between the group of eutrophic newborns from MCDA twin pregnancies with sIUGR (sIUGR Group) and the group of newborns from uncomplicated MCDA twin pregnancies (Uncomplicated Group).

	sIUGR Group	Uncomplicated Group	*p* Value Adjusted *
**Hospitalization in intensive care, n/N (%)**	36/57 (63%)	66/270 (24%)	<0.001
**Length of hospital stay, days, median (min–max)**	4.5 (1–70)	4 (1–39)	0.51
**Death, n/N (%)**	2/57 (4%)	7/270 (3%)	0.81
Age at death, days, median (min–max)	14 (3–25)	7 (4–13)	
**Respiratory complications:**			
Need for ventilatory support, n/N (%)	39/57 (68%)	69/270 (26%)	<0.001
Duration of ventilatory support, days, median (min–max)	6 (1–71)	5.5 (1–55)	0.99
Hyaline membrane disease, n/N (%)	33/57 (58%)	64/270 (24%)	0.02
Bronchopulmonary dysplasia, n/N (%)	3/57 (5%)	7/270 (3%)	0.34
**Sepsis, n/N (%)**	5/55 (9%)	9/187 (5%)	0.29
Early-onset sepsis, n/N (%)	0/55 (0%)	5/187 (3%)	
Late-onset sepsis, n/N (%)	5/55 (9 %)	4/185 (2%)	
**Necrotizing enterocolitis, n/N (%)**	4/57 (7%)	6/270 (2%)	0.22
**Abnormal Auditory Evoked Potentials, n/N (%)**	1/31 (3%)	6/95 (6%)	0.34
**Abnormal indirect ophthalmoscopy, n/N (%)**	3/19 (16%)	6/35 (17%)	0.92
**Abnormal electroencephalogram, n/N (%)**	6/43 (14%)	3/67 (4%)	0.23
**Abnormal brain MRI, n/N (%)**	9/16 (56%)	9/23 (39%)	0.32
Grade III or IV IVH, n/N (%)	1/16 (6%)	1/23 (4%)	0.52
PVL, n/N (%)	2/16 (13%)	0/23 (0%)	0.05
Ventricular dilation, n/N (%)	2/16 (13%)	1/23 (4%)	0.52
**Breastfeeding at discharge, n/N (%)**	34/53 (64%)	98/179 (55%)	0.22
**Composite morbidity and mortality criterion, n/N (%) (including neonatal death, grade III or IV IVH, PVL, BPD, and stage II or III NEC)**	6/57 (11%)	14/270 (5%)	0.18

Data are presented as N (%) unless stated differently. * *p* value adjusted for gestational age, birth weight, and fetal sex.

**Table 3:** Comparison of demographic and neonatal data in patients not lost to follow-up for ASQ analysis between the group of eutrophic newborns from MCDA twin pregnancies with sIUGR (sIUGR Group) and the group of newborns from uncomplicated MCDA pregnancies (Uncomplicated Group).

	sIUGR Group N = 38	Uncomplicated Group N = 67	*p* Value
**Age, years, median (min–max)**	31 (22–40)	30 (19–44)	0.60
**Gravidity, n, median (min–max)**	2 (1–5)	3 (1–7)	0.04
**Parity, n, median (min–max)**	1 (0–4)	1 (0–3)	0.19
**BMI, kg/m^2^, median (min–max)**	21.3 (17.4–43)	24 (16.3–45)	0.02
**Fetal sex, n (%)**			0.12
Male	18	40	
Female	20	27	
**Antenatal corticosteroid therapy, n (%)**			
Not administered	8	36	0.002
Incomplete	0	0	
Complete	30	31	
**Mode delivery, n (%)**			<0.001
Vaginal delivery	6/38 (16%)	37/67 (55%)	
Cesarean section	32/38 (84%)	30/67 (45%)	
**Indication for birth, n (%)**			
Spontaneous	9	30	
Abnormal fetal heart rate	16	1	
Doppler abnormalities	12	3	
Growth restriction	8	3	
Other	3	24	
**Gestational age at birth, weeks of amenorrhea, median (min–max)**	34 (26–35)	36 (27–37)	<0.001
**Birth weight, grams, mean** ± **SD**	1986 ± 456	2384 ± 519	<0.001
**Number of newborns, n**	**38**	**134**	
**Head circumference at birth, centimeters, mean** ± **SD**	30 ± 1.8	31.4 ± 2.3	0.002
**Length at birth, centimeters, mean** ± **SD**	42.5 ± 3.2	44.7 ± 3.5	0.006
**Growth discordance at birth, %, mean** ± **SD**	25 ± 27	6 ± 7	<0.001
**Birth weight percentile, %, mean** ± **SD**	26.5 ± 12	36.7 ± 24.5	0.55
**Apgar score, median, (min–max)**			
1 min	9 (0–10)	9.5 (0–10)	0.12
5 min	10 (5–10)	10 (6–10)	0.11
10 min	10 (8–10)	10 (7–10)	0.004
**Apgar score < 7, n/N (%)**			
1 min	3	18	0.3
5 min	3	5	0.23
10 min	0	0	1
**pH at birth, median, (min–max)**	7.34 (7.19–7.4)	7.3 (7.03–7.5)	0.002
PH < 7.15, n/N (%)	0	11	0.06
**Lactate at birth, mmol/L,** median, (min–max)	2.3 (1.3–5.5)	2.9 (1–6.9)	0.15

**Table 4:** Comparison of ASQ-24 results at 2 years between the group of eutrophic newborns from MCDA twin pregnancies with sIUGR (sIUGR Group) and the group of newborns from uncomplicated MCDA twin pregnancies (Uncomplicated Group).

	sIUGR Group	Uncomplicated Group	Adjusted *p* Value **
**ASQ score**			
Median (IQR)	252 (226–264)	240 (220–260)	0.26
ASQ < 220, n (%)	8/38 (21%)	33/134 (25%)	0.65
ASQ < threshold, n (%) *	11/38 (29%)	47/134 (35%)	0.51
**Number of impaired domains, n (%)**			0.32
0	32 (80%)	92 (69%)	
1	6 (15%)	30 (22%)	
2	1 (3%)	12 (9%)	
3	1 (3%)	0 (0%)	
4 or 5	0	0	
**At least one impaired domain, n (%)**	8/38 (21%)	42/134 (31%)	0.21
**By domain, n (%)**			
Communication	4/38 (11%)	19/134 (14%)	0.59
Gross motor skills	1/38 (3%)	5/134 (4%)	0.68
Fine motor skills	1/38 (3%)	6/134 (4%)	0.44
Problem solving	1/38 (3%)	0/134 (0%)	0.21
Social skills	4/38 (11%)	24/134 (18%)	0.30

Data are presented as N (%) unless stated differently. * Threshold: ASQ < 220 and at least one impaired domain; ** ***p*-value adjusted for gestational age, birth weight, and fetal sex.**

**Table 5:** Comparison of ASQ at 2 years by gestational age between the group of eutrophic newborns from MCDA twin pregnancies with sIUGR (sIUGR Group) and the group of newborns from uncomplicated MCDA twin pregnancies (Uncomplicated Group).

	26–31 Weeks GA			32–34 Weeks GA			>35 Weeks GA		
	sIUGR Group	Uncomplicated Group	*p* Value	sIUGR Group	Uncomplicated Group	*p* Value	sIUGR Group	Uncomplicated Group	*p* Value
**ASQ score,** median (IQR)	250 (220–264)	240 (230–250)	0.37	245 (219–262)	232 (209–245)	0.5	260 (242–265)	240 (220–261)	0.09
ASQ < 220, n (%)	3/10 (30%)	2/10 (20%)	0.84	4/16 (25%)	7/24 (29%)	0.92	1/12 (8 %)	24/100 (24%)	0.17
ASQ < threshold, n (%)	3/10 (30%)	2/10 (20%)	0.84	5/16 (31%)	13/24 (54%)	0.15	1/11 (8%)	24/100 (24%)	0.17
**Number of impaired domains, n (%)**									
**0**	9 (90%)	10 (100%)	1	12 (75%)	13 (54%)	0.78	9 (75%)	69 (69%)	0.38
**1**	1 (10%)	0		2 (13%)	9 (38%)		3 (25%)	21 (21%)	
**2**	0	0		1 (6%)	2 (8%)		0	10 (10%)	
**3**	0	0		0	0		0	0	
**4 or 5**	0	0		0	0		0	0	
**At least one impaired domain, n (%)**	1/10 (10%)	0/10 (0%)	0.49	4/16 (25%)	11/24 (46%)	0.15	3/12 (25%)	31/100 (31%)	0.57
Communication	1	0	0.49	2/16 (13%)	4/24	0.68	1/12	15/100	0.66
Gross motor skills	0	0	1	1/16 (6%)	3/24	0.64	0/12	2/100	0.88
Fine motor skills	0	0	1	0/16	1/24	1	1/12	6/100	0.94
Problem-solving	0	0	1	1/16 (6%)	0/24	0.36	1/12	1/100	1
Social and individual aptitudes	0	0	1	3/16 (19%)	6/24	0.71	1/12	18/100	0.37

**Table 6:** Comparison of neonatal data of children from MCDA twin pregnancies with IUGR: eutrophic child compared to their IUGR co-twin.

	Eutrophic Twin	IUGR Twin	*p* Value
**Hospitalization in intensive care, n/N (%)**	36/57 (63%)	30/57 (53%)	0.35
Duration in intensive care, days, median (min–max)	4.5 (1–70)	5 (1–63)	0.75
**Duration of hospitalization, days, median (min–max)**	25 (0–113)	23 (3–113)	0.96
**Death, n/N (%)**	2/57 (4%)	4/57 (7%)	0.24
Age at death, days, median (min–max)	14 (3–25)	14.5 (6–100)	
**Respiratory complications:**			
Need for ventilatory support, n/N (%)	39/57 (68%)	34/57 (60%)	0.44
Duration of ventilatory support, days, median (min–max)	6 (1–71)	6 (1–86)	0.46
Hyaline membrane disease, n/N (%)	33/57 (58%)	26/57 (46%)	0.18
Bronchopulmonary dysplasia, n/N (%)	3/57 (5%)	4/57 (7%)	0.85
**Infectious complications**			
**Sepsis, n/N (%)**	5/55 (9%)	7/54 (13%)	0.5
Early-onset, n/N (%)	0	0	
Late-onset, n/N (%)	5	7	
**Necrotizing enterocolitis, n/N (%)**			0.37
Stage I, n/N (%)	4/57 (7%)	8/57 (14%)	
Stage II, n/N (%)	1	3	
Stage III, n/N (%)	3	3	
Stage IV, n/N (%)	0	2	
**Neurosensory complications:**			
**Auditory evoked potential abnormalities, n/N (%)**	1/31 (3%)	1/34 (3%)	0.71
**Fundus abnormalities, n/N (%)**	3/19 (16%)	2/17 (12%)	0.72
**Electroencephalogram abnormalities, n/N (%)**	6/43 (13%)	2/46 (4%)	0.12
**Abnormalities on brain MRI, n/N (%)**	9/16 (56%)	3/18 (17%)	0.001
IVH grade III or IV, n/N (%)	1/16 (6%)	0/18 (0%)	0.17
PVL, n/N (%)	2/16 (13%)	1/18 (6%)	0.47
Ventricular dilation, n/N (%)	2/16 (13%)	0/18 (0%)	0.09
**Composite morbidity–mortality criterion, n/N (%)** **(including neonatal death, grade III or IV IVH, PVL, BPD and stage II or III NEC)**	6/57 (11%)	9/57 (16%)	0.58

**Table 7:** Comparison of 2-year data in monochorionic diamniotic pregnancies with IUGR: eutrophic child compared to their IUGR co-twin.

	Eutrophic N = 38	IUGR N = 19	*p* Value
**ASQ score**			
Median (IQR)	255 (229–270)	245 (212–260)	0.53
ASQ < 220, n (%)	8/38 (21%)	6/19 (32%)	0.70
ASQ < threshold, n (%)	11/38 (29%)	8/19 (42%)	0.60
**Number of impaired domains, n (%)**			0.55
0	30	12 (6%)	
1	6	5 (26%)	
2	1	2 (11%)	
3	1	0	
4 or 5	0	0	
At least one altered domain, n (%)	8/38 (21%)	7/19 (37%)	0.38
By domain, n (%)			
Communication	4	5	
Gross motor skills	1	1	
Fine motor skills	1	0	
Problem-solving	1	0	
Social and individual aptitudes	4	3	

## Data Availability

The datasets that generated and/or analyzed during the current study are not publicly available due to the fact that the data belong to the Assistance Publique Hopitaux de Marseille. However, datasets are available from the sponsor (promotion.interne@ap-hm.fr) on reasonable request and after signing a contract pertaining to the provision of data and/or results.
